# Haplotypes of *ABCB1* 1236C >T (rs1128503), 2677G >T/A (rs2032582), and 3435C >T (rs1045642) in patients with bullous pemphigoid

**DOI:** 10.1007/s00403-018-1842-8

**Published:** 2018-06-09

**Authors:** Mariola Rychlik-Sych, Małgorzata Barańska, Michał Dudarewicz, Jadwiga Skrętkowicz, Agnieszka Żebrowska, Anna Woźniacka, Jacek Owczarek, Daria Orszulak-Michalak, Elżbieta Waszczykowska

**Affiliations:** 1 Department of Pharmacogenetics, Medical University of Lodz, Muszyńskiego 1, 90-151 Lodz, Poland; 2 Department of Dermatology and Venereology, Medical University of Lodz, Plac Hallera 1, 90-647 Lodz, Poland; 3 Department of Biopharmacy, Medical University of Lodz, Muszyńskiego 1, 90-151 Lodz, Poland

**Keywords:** *ABCB1* polymorphism, Haplotype analysis, P-glycoprotein, Bullous pemphigoid

## Abstract

Bullous pemphigoid (BP) constitutes the most prevalent disease in the group of bullous dermatoses with the autoimmune background. Some authors suggest that certain cytokines (IL-2, IFN-γ) may be transported by P-glycoprotein (P-gp), the product of the *ABCB1* gene. *ABCB1* polymorphism might affect not only the effectiveness of treatment with drugs that are P-gp substrates but also contribute to the development of diseases, including BP. In the present work, we resolved to conduct a haplotype analysis of *ABCB1* in patients with BP and to answer the question of whether any of the haplotypes are able to affect the incidence of this entity. The study involved 71 patients with BP and 100 healthy volunteers. Determination of polymorphisms 1236C > T and 3435C > T in *ABCB1* was carried out with the PCR–RFLP (Polymerase Chain Reaction–Restriction Fragment Length Polymorphism) method. The 2677G > T/A *ABCB1* polymorphism was analyzed with the allele-specific PCR method. It was observed that the 1236T-2677G-3435T haplotype occurred with a statistically significantly lower frequency in patients with BP than in controls (1.4 vs. 10.0%). Carriers of this haplotype were also shown to have had a low relative risk for BP (OR = 0.13, *p* = 0.003). Haplotype analysis of *ABCB1* conducted in patients with BP demonstrated that the 1236T-2677G-3435T haplotype may protect against development of this entity.

## Introduction

Bullous pemphigoid (BP) belongs to a class of autoimmune diseases resulting from a disorder of tolerance of one’s own antigens. It is characterized by the presence of antibodies against hemidesmosomes and basement membrane components. In BP, one can find inflammatory infiltrations in the dermis, deposits of immunoglobulins (primarily IgG), and constituents of complement (primarily C3) along the basement membrane zone (BMZ), and circulating autoantibodies (IgG). Main autoantigens, in this case, are hemidesmosomal proteins, denoted as BPAG1 (BP230) and BPAG2 (BP180) [21, 39]. Binding of autoantibodies to them results in an activation of keratinocytes, mast cells, eosinophils and neutrophils, and C5 of the complement. Subsequently, IL-6, IL-8, and matrix metalloproteinases are released, which leads to destruction of basement membrane components, anchoring fibers, and to the formation of blisters [33, 46].

There is evidence that in addition to immunological factors, genetic factors play a significant role in the etiology and pathogenesis of BP. It has been shown that an association between BP and the following alleles exists: HLA-DQβ1*0301 (in a Caucasian population) HLA-DRB1*04, DRB1*1101, and DQB1*0302 (in a Japanese population) [21, 25, 44]. Some authors have recently pointed to the relevance of mitochondrially encoded ATP synthase 8 gene (*MT-ATP8*) and Fcgamma-receptor IIIa gene (CD16) polymorphisms in the development of BP [12, 40].

BP usually occurs in individuals over 65 year of age, though the main group of patients is comprised of those over 80 year of age. Therefore, the treatment of BP requires a great deal of caution. An ongoing therapy may exacerbate the course of concomitant diseases or lead to new ones. Currently, in the treatment of BP, glucocorticosteroids (GCS), tetracyclines, and methotrexate are used. Many of them are transported by P-glycoprotein (P-gp), which may affect their bioavailability and concentration at the site of action [16].

P-gp is a membrane transporter responsible for the regulation of the flow of both endogenous substances and xenobiotics between cells and their surroundings. An alteration in activity and structure of P-gp resulting from the presence of genetic polymorphisms may lead to lower treatment efficacy [30]. Gene *ABCB1* (ATP binding cassette subfamily B member 1), which encodes P-gp, is found in the long arm of chromosome 7 (7q21.12). So far, over 50 single-nucleotide polymorphisms (SNP) and three insertion/deletion polymorphisms within *ABCB1* have been described. Three polymorphisms among them seem to be of the highest biological importance: a change at 1236 in exon 12 (NM_000927.4:c.1236C > T, rs1128503), at 2677 in exon 21 (NM_000927.4:c.2677G > A/T, rs2032582), and at 3435 in exon 26 (NM_000927.4:c.3435C > T, rs1045642). In a study by Pawlik et al. [29] it was demonstrated that *ABCB1* polymorphisms (3435C > T and 2677G > T/A) influenced the secretion of some cytokines (IL-2, IL-4, IFN-γ, and TNF-α) in cell cultures treated with methotrexate and dexamethasone.

Genetic polymorphisms of P-gp might affect not only the effectiveness of treatment with drugs that are P-gp substrates, but also contribute to the development of diseases [2, 5, 9, 27, 32, 44, 45].

As there is a hypothesis that three *ABCB1* polymorphisms (1236C > T, 2677G > T/A, and 3435C > T) are co-inherited within a haplotype, in the present work, we resolved to conduct a haplotype analysis of *ABCB1* in patients with BP and to answer the question of whether any of the haplotypes are able to affect the incidence of this entity.

## Materials and methods

### Study population

The study involved a total of 171 individuals, including 71 patients with BP and 100 healthy volunteers who constituted the control group. The patients were treated in the Department of Dermatology and Venereology at Medical University of Lodz, Poland. The age of BP patients ranged from 29 to 92 years [mean ± standard deviation (SD), 66.3 ± 15.0; 47 females, 24 males] and that of healthy volunteers from 19 to 75 years (mean ± SD, 36.9 ± 12.5; 45 females, 55 males). All patients were at the active stage of the disease, had not yet been administered any drugs (systemic or topical), and had an average score of BPDAI (Bullous Pemphigoid Disease Activity Index) ± SD being 39 ± 16. The histopathologic features, according to Ackerman, were fully developed in all cases. The specimens revealed in all cases neutrophilic, eosinophilic, and lymphocytic infiltrates in the dermis, and in most cases—subepidermal blisters. In all of the patients, a direct immunofluorescence (DIF) test showed IgG and/or C3 linear deposits along BMZ. In 1 M NaCl split tests, deposits were observed on the epidermal part of the blister or on the epidermal and dermal part of the split. Using indirect immunofluorescent test, circulating IgG antibodies were detected in the patients’ sera during incubation with monkey’s esophagus [26]. The antibodies were found in 70% cases, in titers ranging from 1:80 to 1:320 (median 160) (Table 1). Design of the study has been given a positive opinion by Bioethics Committee on Research in Humans at the Medical University of Lodz.

**Table 1:** Demographic and clinical characteristics of patients with BP and controls

Variable	Patients with BP (*N* = 71)	Controls (*N* = 100)
Sex, *n* (%)		
Female	47 (66.2%)	45 (45%)
Male	24 (33.8%)	55 (55%)
Age (mean ± SD)	66.3 ± 15.0	36.9 ± 12.5
Age group, *n* (%)		
< 20	–	5 (5%)
21–40	6 (8.4%)	61 (61%)
41–60	18 (25.3%)	29 (29%)
61–80	35 (49.3%)	5 (5%)
81–100	12 (17.0%)	–
BDAI (mean ± SD)	39 ± 16	–
VAS (itch)	4–10 (median 8)	–
Anti BMZ antibodies	1:80 to 1:320 (median 60)	–

*BDAI* bullous pemphigoid disease activity index, *VAS* visual analogue scale, *BMZ* basement membrane zone

### Genotyping

Genomic DNA was extracted from peripheral blood leukocytes using Gene MATRIX Quick Blood DNA Purification Kit (EURx Ltd., Poland) according to the manufacturer’s protocol.

Analysis of the 1236C > T polymorphism in *ABCB1* was carried out with the use of the PCR–RFLP (*Polymerase Chain Reaction–Restriction Fragment Length Polymorphism*) method, which was based on the procedure by Cascorbi et al. [7]. The application of the *HaeIII* restriction endonuclease (EURx Ltd., Poland) enabled the detection of the wild-type 1236C allele and the variant 1236T allele in the study participants. Patients with pemphigoid and healthy volunteers were divided into genotypes: 1236CC (wild-type homozygotes), 1236CT (heterozygotes), and 1236TT (variant homozygotes).

Analysis of the 2677G > T/A polymorphism in *ABCB1* was made with the allele-specific PCR method based on the procedure submitted by Kurzawski et al. [19]. The 2677G > T/A polymorphism was determined using a set of primers specific to the *ABCB1* gene fragments flanking position 2677. The wild-type allele (2677G) and two variant alleles (2677T and 2677A) were distinguished. Both patients with BP and healthy volunteers were classified by genotype: 2677GG (wild-type homozygotes), 2677GT, 2677GA, 2677TA (heterozygotes), and 2677TT, 2677AA (variant homozygotes).

Determination of the 3435C > T polymorphism in *ABCB1* was performed with the use of the PCR–RFLP method based on the procedure by Siegmund et al. [34]. Using the *Sau3AI* restriction enzyme (EURx Ltd., Poland) enabled the division of individuals according to the presence of the wild-type allele (3435C) and the variant one (3435T). Among patients with BP and healthy volunteers, there were genotypes 3435CC (wild-type homozygotes), 3435CT (heterozygotes), and 3435TT (variant homozygotes).

### Statistical analysis

The distribution of particular alleles and genotypes in patients with BP was compared to the distribution in the control group of healthy volunteers. Accordance of *ABCB1* genotypes to Hardy–Weinberg equilibrium was checked using a *χ*^2^ test. To determine differences in the frequencies of particular *ABCB1* haplotypes between both groups, first, a permutation test and then a *χ*^2^ test were applied. Subsequently, it was assessed the risk for BP expressed as odds ratio (OR), among carriers of particular *ABCB1* alleles, genotypes, and haplotypes. Calculations were made with the use of *PHASE v2.1* software and with the use of *STATISTICA 12* data analysis software system (StatSoft Ltd., Krakow, Poland). The program *PHASE* implements a Bayesian statistical method for reconstructing haplotypes from genotype data [35, 36]. As there was a significant difference in the distribution of particular *ABCB1* haplotypes between cases and controls (*p* = 0.01 for a permutation test, which was pre-installed), we wanted to know the influence of particular haplotypes on the BP risk. Therefore, output data were converted to figures according to the sample haplotype frequencies generated with *PHASE*, which is some limitation of this study. Then, logit regression in the non-linear estimation module of *STATISTICA* was applied to determine odds ratio for BP. A significance level of *p* < 0.05 was considered as statistically relevant.

## Results

We have stated that *ABCB1* genotypes at positions 1236, 2677, and 3435 were in accordance with the Hardy–Weinberg equilibrium both in the BP group and in the control group (data not shown).

The frequencies of particular *ABCB1* alleles and genotypes for 1236C > T, 2677G > T/A and 3435C > T polymorphisms in the group of BP patients and in the control group are depicted in Table 2. An analysis of individual *ABCB1* polymorphisms has shown that only one of them, i.e., at position 2677, may affect morbidity to BP. It was observed that 2677TT and 2677TA genotypes occurred with statistically significantly higher frequency in BP patients compared with the control group (32.4 vs. 18.0%; 9.0 vs. 3.0%, respectively). Assessed relative risk for BP was over fourfold greater in carriers of the 2677TA genotype (OR = 4.69, 95% CI 1.22–18.01, *p* = 0.0326) and over twofold greater in carriers of the 2677TT genotype (OR = 2.18, 95% CI 1.07–4.45, *p* = 0.0298). Variant alleles were more frequent in the BP group than in the control group [56.3 vs. 36.5% (*p* = 0.0003) for the 2677T allele and 9.2 vs. 4.0% (*p* = 0.0504) for the 2677A allele]. Decreased risk for BP was found in carriers of the 2677GG genotype and the wild-type 2677G allele (OR = 0.25, 95% CI 0.11–0.54, *p* = 0.0002; OR = 0.36, 95% CI 0.23–0.56, *p* = 0.00001, respectively). The frequency of individuals with the 2677GG genotype was greater in the control group than in the BP group (40.0 vs. 14.1%, respectively), which may suggest a protective role of this genotype against BP.

**Table 2:** Genotypes and alleles frequency for analyzed SNPs in *ABCB1* in the group of BP patients and the control group

Genotypes ABCB1	BP *N* (%)m *N* = 71	Controls *N* (%), *N* = 100	*p* value	OR (95% CI)	Alleles ABCB1	BP *N* (%), *N* = 142	Controls *N* (%), *N* = 200	*p* value	OR (95% CI)
1236C > T					1236C > T				
CC CT TT	18 (25.4) 43 (60,6) 10 (14.1)	36 (36.0) 46 (46.0) 18 (18.0)	0.1399 0.0603 0.4954	0.60 (0.31–1.18) 1.80 (0.97–3.34) 0.75 (0.32–1.73)	C T	79 (55.6) 63 (44.4)	118 (59.0) 82 (41.0)	0.5348 0.5348	0.87 (0.56–1.35) 1.15 (0.74–1.77)
2677G > T/A					2677G > T/A				
GG GT GA TT TA	10 (14.1) 25 (35.2) 4 (5.6) 23 (32.4) 9 (12.7)	40 (40.0) 34 (34.0) 5 (5.0) 18 (18.0) 3 (3.0)	0.0002* 0.8696 0.8693 0.0298* 0.0326*	0.25 (0.11–0.54) 1.05 (0.56-2.00) 1.13 (0.29–4.38) 2.18 (1.07–4.45) 4.69 (1.22–18.01)	G T A	49 (34.5) 80 (56.3) 13 (9.2)	119 (59.5) 73 (36.5) 8 (4.0)	0.00001* 0.0003* 0.0504	0.36 (0.23–0.56) 2.24 (1.45–3.48) 2.42 (0.97-6.00)
3435C > T					3435C > T				
CC CT TT	17 (23.9) 31 (43.7) 23 (32.4)	20 (20.0) 45 (45.0) 35 (35.0)	0.5372 0.8623 0.7229	1.26 (0.61–2.62) 0.95 (0.51–1.75) 0.89 (0.47–1.70)	C T	65 (45.8) 77 (54.2)	85 (42.5) 115 (57.5)	0.5476 0.5476	1.14 (0.74–1.76) 0.88 (0.57–1.35)

*BP* Bullous pemphigoid, *95% CI* 95% confidence interval, OR: odds ratio

*p* Level of statistical significance, *Statistically significant differences (*p* < 0.05)

The frequencies of *ABCB1* haplotypes for polymorphisms 1236C > T, 2677G > T/A, and 3435C > T in the group of patients with pemphigoid and in the control group are presented in Table 3. These frequencies were directly obtained with *PHASE v.2.1*. In the BP group, the haplotype 1236T-2677T-3435T predominated (33.8%), whereas in the control group, the haplotype 1236C-2677G-3435C was the most frequent (32.0%). It was observed that the haplotype 1236T-2677G-3435T occurred with a statistically significantly lower frequency in patients with BP than in controls (1.4 vs. 10.0%). Carriers of this haplotype were also shown to have had a low relative risk for BP (OR = 0.13, 95% CI 0.03–0.56, *p* = 0.003).

**Table 3:** Frequency of *ABCB1* haplotypes in the group of BP patients and the control group, and the risk for BP

Haplotypes C1236T-G2677A/T-C3435T	BP *N* (%)	Control group *N* (%)	*p* value	OR (95% CI)
C-G-C	33 (23.2)	64 (32.0)	0.0765	0.64 (0.39–1.05)
C-G-T	11 (7.8)	29 (14.5)	0.0555	0.50 (0.24–1.03)
C-T-C	13 (9.2)	8 (4.0)	0.0504	2.42 (0.97–6.00)
C-T-T	13 (9.2)	9 (4.5)	0.0838	2.14 (0.89–5.15)
C-A-C	5 (3.5)	3 (1.5)	0.3923	2.40 (0.56–10.20)
C-A-T	4 (2.8)	4 (2.0)	0.8970	1.42 (0.35–5.78)
T-G-C	4 (2.8)	6 (3.0)	0.8207	0.94 (0.26–3.38)
T-G-T	2 (1.4)	20 (10.0)	0.003*	0.13 (0.03–0.56)
T-T-C	5 (3.5)	3 (1.5)	0.3923	2.40 (0.56–10.20)
T-T-T	48 (33.8)	53 (26.5)	0.1446	1.42 (0.89–2.26)
T-A-C	4 (2.8)	1 (0.5)	0.1929	5.77 (0.64–52.17)
N-N-N vs. C-G-C			0.0765	1.55 (0.95–2.54)
T-T-T vs. C-G-C			0.0534	1.76 (0.99–3.12)

*BP* bullous pemphigoid, *95% CI* 95% confidence interval, *OR* odds ratio, *N* variant allele

*p* - level of statistical significance, *Statistically significant differences (*p* < 0.05)

The difference in frequencies of two other *ABCB1* haplotypes was at the border of statistical significance, which is noteworthy. The 1236C-2677G-3435T haplotype was less frequent in the group of BP patients than in the control group (7.8 vs. 14.5%); hence, the relative risk of developing BP was low (OR = 0.50, 95% CI 0.24–1.03, *p* = 0.0555). On the contrary, the 1236C-2677T-3435C haplotype occurred more frequently in patients with BP than in controls (9.2 vs. 4.0%). The relative risk for BP was demonstrated to have been over twofold greater among carriers of this haplotype (OR = 2.42, 95% CI 0.97–6.00, *p* = 0.0504).

We found that carriers of the T-T-T haplotype had an increased risk for BP (OR = 1.76, 95% CI 0.99–3.12) when compared with carriers of the C-G-C haplotype, which consists of wild-type alleles. The association was also at the border of statistical significance (*p* = 0.0534; Table 3).

## Discussion

P-gp is a transmembrane transporter made up of 1280 amino acids, with activity depending on ATP hydrolysis. Its localization suggests a relevant role in protection of cells against toxic compounds. The highest level of P-gp expression was observed in enterocytes in the small and large intestine (where P-gp diminishes the absorption of toxins from food), hepatocytes, epithelial cells of kidney proximal tubules (where it participates in the elimination of toxins and metabolites with urine and gall), and in placental cells. P-gp is expressed to a lesser degree in the endothelium of blood vessels in the central nervous system and testicles, where it co-creates blood–brain and blood–testicle barriers [3, 31].

P-gp is thought to reduce the toxic impact of xenobiotics on the body. There are many drugs, which are also xenobiotics, in a substrate spectrum of P-gp. These include cancer chemotherapeutic drugs, cardiovascular system drugs, HIV protease inhibitors, immunosuppressives, antibiotics, adrenocorticosteroids, opioid analgesics, and antipsychotic agents [19]. The elimination of a drug from the body, to which P-gp contributes, leads to the diminishing of the drug concentration at the site of action, thus decreasing its therapeutic efficacy. It was observed that not only environmental factors (temperature shock, UV light, free radicals, some metals), but also variations within *ABCB1* affect the expression and activity of P-gp. Consequently, alterations in drug bioavailability may occur [1].

So far, over 50 single-nucleotide polymorphisms in *ABCB1* have been identified. The first one was the 2677G > T/A polymorphism described by Mickley et al. [24], which results in a change of the P-gp amino acid sequence (Ala→Ser/Thr). In 2000, Hoffmeyer et al. [13] reported the presence of a silent mutation in exon 26 at position 3435 with a functional effect (transition C → T). At the time, it was noted that individuals with the 3435CC genotype had a twofold greater expression of P-gp in duodenal epithelium in comparison with the variant homozygotes (3435TT). Although a silent polymorphism at 3435 does not alter the amino acid sequence of P-gp, the resulting change of codon may influence the folding of mRNA, the process of translation and, secondarily, P-gp activity [6, 17]. The influence of the 3435C > T polymorphism on P-gp activity may be explained by linkage disequilibrium between positions 1236 (C → T, silent mutation), 2677 (missense), and 3435 (silent). These three SNP are probably co-inherited within a single haplotype [13].

In addition to having an impact on the expression and activity of P-gp, polymorphism of *ABCB1* may also affect predisposition to diseases and pharmacotherapy, which involves administration of agents that are substrates of P-gp.

In the present work, analyses of these three SNP in *ABCB1* have shown that only one of them may affect the risk for BP, i.e., SNP at position 2677. Greater risk was found in carriers of two genotypes: 2677TA (OR = 4.69, 95% CI 1.22–18.01, *p* = 0.0326) and 2677TT (OR = 2.18, 95% CI 1.07–4.45, *p* = 0.0298). Little risk for BP, in turn, was observed in individuals with the 2677GG genotype (OR = 0.25, 95% CI 0.11–0.54, *p* = 0.0002). Increased risk for BP was associated with alleles: 2677T (OR = 2.24, 95% CI 1.45–3.48, *p* = 0.0003) and 2677A (OR = 2.42, 95% CI 0.97–6.0, *p* = 0.0504), and the presence of the 2677G allele decreased the risk markedly (OR = 0.36, 95% CI 0.23–0.56, *p* = 0.00001).

We also demonstrated that the 1236T-2677G-3435T haplotype might protect against pemphigoid. This haplotype was observed to be statistically significantly less frequent in the group of BP patients than in the control group (1.4 vs. 10.0%, Table 3). The 1236C-2677G-3435T haplotype could have safeguarded individuals against BP as well. The association was not statistically relevant, though (OR = 0.5, 95% CI 0.24–1.03, *p* = 0.0555). Contrary to these findings, the 1236C-2677T-3435C haplotype might have contributed to BP development. The frequency of the C-T-C haplotype was greater in patients with BP than in healthy controls (9.2 vs. 4.0%) and the association was found at the border of statistical significance (OR = 2.42, 95% CI 0.97–6.00, *p* = 0.0504).

According to the literature, *ABCB1* polymorphism has been determined in numerous disease entities. A haplotype analysis within *ABCB1* performed, i.a., in neoplastic and neurological diseases turned out to be a useful tool in terms of evaluation of the morbidity and the effectiveness of treatment [2, 4, 20, 22, 23, 27, 42, 43, 45].

In available literature, one can find papers on the role of *ABCB1* polymorphisms in dermatologic diseases with an autoimmune background, which pemphigoid is classified as [5, 8, 9, 11, 37, 38]. Autoimmune diseases belong to chronic disorders with unknown etiology, characterized by a broad spectrum of immune system abnormalities. Since P-gp participates in the transport of cytokines (e.g., IL-2 and IFN-γ), P-gp may be involved in ongoing immunologic processes in the body [10, 28].

A haplotype analysis of *ABCB1*, which had been conducted in patients with systemic sclerosis (SSc) in Poland, revealed a statistically significantly higher frequency of the 1236C-2677G-3435T haplotype in the SSc group in comparison with the control group (25 vs. 15%, *p* = 0.032). Consequently, the study determined that individuals with the haplotype have a nearly twofold higher risk of developing SSc (OR = 1.85, 95% CI 1.05–3.25, *p* = 0.032) [5].

In a study involving patients with systemic lupus erythematosus (SLE) in Brazil, it was shown that polymorphisms 1236C > T, 2677G > T/A, and 3435C > T do not alter the risk for this disease. It was observed that haplotype 1236C-2677G-3435C and haplotype 1236T-2677T-3435T were the most frequent, both in the SLE group and in the control group (44.9 and 28.2%; 44.8 and 27.0%, respectively). Moreover, allele 2677A is likely to have an impact on clinical features of SLE [11].

Determination of *ABCB1* polymorphisms may be helpful when treating autoimmune diseases, including pemphigoid. Administration of glucocorticosteroids and immunosuppressive agents ameliorates the course of the diseases; however, a major number of patients do not respond to treatment and develop drug resistance. One of the drugs which is used in the treatment of autoimmune diseases, including rheumatoid arthritis, connective tissue diseases, and severe psoriasis, is methotrexate [15]. In patients with pemphigoid, application of methotrexate along with systemic GCS yielded a decreased demand for the latter and lower frequency of adverse drug reactions induced by GCS [18]. This is very important, as studies have pointed out that 40% of individuals treated with oral GCS die within the first year of therapy due to septicemia, pneumonia, cardiovascular disease, or stroke [14]. Therefore, in the treatment of BP, alternative regimens are being used (e.g., involving administration of a tetracycline along with nicotinamide) as well [41]. GCS, immunosuppressive drugs, and tetracyclines are substrates of P-gp; thus, its overexpression can influence outcomes of the treatment [16, 19].

An impact of *ABCB1* polymorphisms on treatment efficacy in patients with rheumatoid arthritis (RA) was demonstrated in a study by Cuppen et al. [8]. The authors noted that carriers of genotypes 2677GT and 2677TT had better responded to intravenous administration of methylprednisolone compared with carriers of the wild-type 2677GG genotype (OR = 6.18, *p* = 0.035). Takatori et al. [37] analyzed patients with RA in a Japanese population and showed that patients with the 3435TT genotype required an increased methotrexate dose in the early phase of treatment in comparison with patients with the wild-type 3435CC genotype (OR = 8.78, *p* = 0.038). However, in a Polish population, Droździk et al. [9] observed that carriers of the 3435TT genotype had the highest percentage of individuals that experienced clinical remission as a result of combined treatment with methotrexate and methylprednisolone. The probability of remission was 4.65-fold greater in patients with the 3435TT genotype as compared to patients with the 3435CC genotype (OR = 4.65, *p* = 0.003).

In patients with psoriasis, 3435C > T polymorphism in *ABCB1* was shown to be associated with response to cyclosporine, which is a substrate of P-gp. The frequency of the variant 3435T allele was found to be significantly higher in non-responders as compared to responders (OR = 2.995, *p* = 0.0075). Further haplotype analysis that covered the positions 1236, 2677, and 3435 only strengthened this association, making the 3435T allele the plausible risk factor [38].

In conclusion, our results indicate that only one out of three analyzed SNP in *ABCB1* may influence the risk for BP. Genotypes 2677TA and 2677TT can predispose to the development of BP, whereas the 2677GG genotype can play a protective role. Haplotype analysis of *ABCB1* demonstrated that the 1236T-2677G-3435T haplotype may protect against BP. In the treatment of this entity, drugs which are substrates of P-gp are administered. Therefore, further studies on polymorphisms 1236C > T, 2677G > T/A, and 3435C > T within *ABCB1* should be pursued in terms of encouraging safe and effective pharmacotherapy. The advanced age of BP patients along with concomitant diseases means that their treatment requires a great deal of caution. Taking into account also the high number of BP patients who do not respond to applied drugs, determination of *ABCB1* polymorphisms may help find the cause of this ineffectiveness in their therapy.
